# Conflicts of Interest: Owens’ Response

**Published:** 2004-10

**Authors:** J. William Owens

**Affiliations:** The Procter and Gamble Company, Central Product Safety, Cincinnati, Ohio, E-mail: owens.jw@pg.com

To clarify comments from Goozner and the Center for Science in the Public Interest (CSPI), I would like to provide the pertinent facts on my compliance with *EHP’s* policy on competing financial interest disclosure.

First, I was on loan or “seconded” from Procter & Gamble (P&G) to the Organisation for Economic Co-operation and Development (OECD) in Paris. The secondment was arranged at the request of the OECD, and I was officially under contract and employed by them from October 2002 through October 2003. I offer the former manager of the department, Herman Köeter; the current manager, Andrew Wagner; and their manager, Robert Visser, as references for these points.

Second, in this approximate period, I was a corresponding or contributing author on 11 relevant publications on assays that were either undergoing formal validation at the OECD or were being developed along similar lines for endocrine mechanisms (Ashby et al. 2002a, 2002b, 2003, 2004; Fang et al. 2003; Kanno et al. 2001, 2003a, 2003b; Owens and Ashby 2002; Owens and Köeter 2003; Owens et al. 2003; Yamasaki et al. 2003). In all cases but one, my P&G address was used.

Third, the one exception involved the article questioned by Goozner (Yamasaki et al. 2003). Kanji Yamasaki of the Chemical Evaluation and Research Institute in Japan was the corresponding author in this case. Because work on this manuscript (Yamasaki et al. 2003) occurred while I was at the OECD, Yamasaki used the OECD affiliation when submitting the manuscript to *EHP*. I simply did not notice the affiliation. If I had, I would have changed it to use my P&G affiliation to be consistent with the other 10 publications during this period.

Last, neither I nor P&G have any financial interest in the publications or their subject matter. Rather, this work was done to progress both development and validation work on safety tests that have potential for use to serve a diverse range of parties, including regulators, industry, and the public. Therefore, I do not see how allegations of a competing financial interest are valid in this case, even considering the oversight on the affiliation.

I hope this fully clarifies the record on this matter and lays the CSPI allegations to rest.
